# Antibacterial activities of the methanol extracts and compounds from *Erythrina sigmoidea against Gram-negative multi-drug resistant phenotypes*

**DOI:** 10.1186/s12906-015-0978-8

**Published:** 2015-12-30

**Authors:** Doriane E. Djeussi, Louis P. Sandjo, Jaurès A. K. Noumedem, Leonidah K. Omosa, Bonaventure T. Ngadjui, Victor Kuete

**Affiliations:** Department of Biochemistry, Faculty of Science, University of Dschang, P.O. Box 67, Dschang, Cameroon; Department of Pharmaceutical Sciences, CCS, Universidade Federal de Santa Catarina, Florianópolis, 88040-900 SC Brazil; Department of Chemistry, School of Physical Sciences, University of Nairobi, P. O. Box, 30197-00100 Nairobi, Kenya; Department of Organic chemistry, Faculty of Science, University of Yaoundé I, Yaoundé, Cameroon

**Keywords:** Antibacterial, *Erythrina sigmoidea*, Compounds, Multidrug resistance, Neobavaisoflavone

## Abstract

**Background:**

In the present study, the methanol extracts from the leaves, as well as compounds namely sigmoidin I (**1**), atalantoflavone (**2**), bidwillon A (**3**), neocyclomorusin (**4**), 6α-hydroxyphaseollidin (**5**) and neobavaisoflavone (**6**) (from the bark extract) were tested for their activities against a panel of Gram-negative bacteria including multi-drug resistant (MDR) phenotypes.

**Methods:**

Broth microdilution method was used to determine the minimum inhibitory concentrations (MICs) and the minimum bactericidal concentrations (MBCs) of the extracts as well as compounds **1**–**6**.

**Results:**

The MIC results indicated that the crude extracts from the leaves and bark of this plant were able to inhibit the growth of 96.3 % of the 27 tested bacteria. Compounds **2**–**6 **displayed selective activities, their inhibitory effects being obtained on 8.3 %, 41.7 %, 58.3 %, 58.3 % and 66.7 % of tested bacteria respectively for **2**, **3**, **5**, **6 **and **4**. The lowest MIC value of 8 μg/mL was obtained with **6 **against *Escherichia coli* ATCC8739, *Enterobacter cloacae* ECCI69, *Klebsiella pneumoniae* KP55, *Providencia stuartii* NAE16 and *Pseudomonas aeruginosa* PA01.

**Conclusion:**

The present study demonstrates that *Erythrina sigmoidea* is a potential source of antibacterial drugs to fight against MDR bacteria. Neobavaisoflavone (**6**) is the main antibacterial consituents of the bark crude extract.

## Background

Medicinal plants have been used since ancient times in the management of human including microbial infections. Approximately 60 % of world’s population still relies on medicinal plants for their primary healthcare [1]. The African mainland has between 40,000-60,000 plant species, of which approximately 35,000 are endemic [2, 3]. Cameroon has a rich biodiversity, with about 8,620 plants species [4]. Several Camerooninan medicinal plants were previously reported for their antibacterial activities against multi-drug resistant Gram-negative bacteria [5–8]. Some of the them include *Beilschmiedia cinnamomea* and *Echinops giganteus* [5], *Beilschmiedia obscura, Pachypodanthium staudtii* and *Peperomia fernandopoiana* [9] or *Capsicum frutescens* [10]. The antimicrobial activities of many secondary metabolites from Cameroonian plants were also reported [11, 12]. In our continuing search of new herbal drug from the Cameroon flora, the present study was designed to demonstrate the antibacterial activity of the extracts and compounds from *Erythrina sigmoidea* Hua (Fabaceae). *Erythrina sigmoidea* is a tree of up to 6 m high, with stems armed with stout found in Senegal, Nigeria, Cameroon, Chad and Central African Republic [13]. The plant is traditionally used as antidotes (venomous stings, bites, etc.), diuretic, febrifuge and to treat arthritis, rheumatism, pulmonary troubles, stomach troubles, infectious diseases and kidney diseases [13]. In the Western Region of Cameroon, the aqueous extracts from leaves, bark and roots are used to treat gastrointestinal infections, venereal diseases and leprosy [14]. Previously phytochemical study this plant led to the isolation of sigmoidin I (**1**), atalantoflavone (**2**), bidwillon A (**3**), neocyclomorusin (**4**), 6α-hydroxyphaseollidin (**5**), and neobavaisoflavone (**6**) [15]. They displayed good cytotoxicity towards drug-sensitive and drug resistant cancer cell line [15]. In addition, they showed low cytotoxicity against the normal AML12 hepatocytes [15].

## Methods

### Plant material and extraction

The leaves and bark of *Erythrina sigmoidea* (Fabaceae) were collected in April 2013 in Bangangté (West Region of Cameroon). The plant was identified by a botanist of the National Herbarium in Yaoundé, Cameroon and compared with voucher kept under the registration number N°24470/HNC.

### Antimicrobial assays

#### Chemicals for antimicrobial assay

**Fig. 1:**
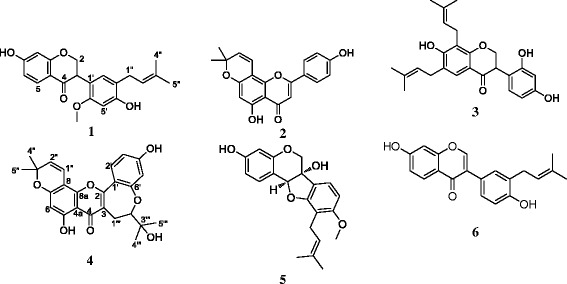
Chemical structures of the compounds isolated from *Erythrina sigmoidea.* sigmoidin I (**1**); atalantoflavone (**2**); bidwillon A (**3**); neocyclomorusin (**4**); 6α-hydroxyphaseollidin (**5**); neobavaisoflavone (**6**)

Compounds isolated from the bark of *Erythrina sigmoidea* included β- sigmoidin I (**1**), atalantoflavone (**2**), bidwillon A (**3**), neocyclomorusin (**4**), 6α-hydroxyphaseollidin (**5**) and neobavaisoflavone (**6**) (Fig. 1). Their isolation and identification were previously reported [15]. Chloramphenicol ≥ 98 % (Sigma-Aldrich, St. Quentin Fallavier, France) was used as reference antibiotics (RA) against Gram-negative bacteria. *p*-Iodonitrotetrazolium chloride ≥ 97 % (INT, Sigma-Aldrich) was used as microbial growth indicator [16, 17].

### Microbial strains and culture media

The studied microorganisms included sensitive and resistant strains of *Escherichia coli* (ATCC8739, AG100, AG100A, AG100A_TET_, AG102, MC4100, W3110), *Enterobacter aerogenes* (ATCC13048, CM64, EA27, EA289, EA294, EA298), *Enterobacter cloacae* (ECCI69, BM47, BM67), *Klebsiella pneumoniae* (ATCC12296, KP55, KP63, K24, K2), *Providencia stuartii* (NEA16, ATCC29916, PS2636, PS299645) and *Pseudemonas aeruginosa* (PA01, PA124) obtained clinically or from the American Type Culture Collection. Their bacterial features are summarized in Table 1. Nutrient agar was used for the activation of the tested bacteria [18].

**Table 1:** Bacterial strains used and their features

Strains	Features and references	
*Escherichia coli*		
ATCC8739	Reference strain	
AG100	Wild-type *E. coli* K-12	[34]
AG100A	AG100 *ΔacrAB*::KAN^R^	[11, 34, 35]
AG100A_TET_	Δ*acrAB* mutant AG100, with over-expressing *acrF* gene; TET^R^	[34]
AG102	Δ*acrAB* mutant AG100, owing *acrF* gene markedly over-expressed; TET^R^	[12, 36]
MC4100	Wild type *E. coli*	[37]
W3110	Wild type *E. coli*	[37, 38]
*Enterobacter aerogenes*		
ATCC13048	Reference strains	
CM64	CHL^R^ resistant variant obtained from ATCC13048 over-expressing the AcrAB pump	[39]
EA27	Clinical MDR isolate exhibiting energy-dependent norfloxacin and chloramphenicol efflux with KAN^R^ AMP^R^ NAL^R^ STR^R^ TET^R^	[40, 41]
EA289	KAN sensitive derivative of EA27	[42]
EA294	EA289 a*crA::*KAN^R^	[42]
EA298	EA 289 *tolC::*KAN^R^	[42]
*Enterobacter cloacae*		
ECCI69	Clinical MDR isolates, CHL^R^	[5]
BM47	Clinical MDR isolates, CHL^R^	[5]
BM67	Clinical MDR isolates, CHL^R^	[5]
*Klebsiella pneumoniae*		
ATCC12296	Reference strains	
KP55	Clinical MDR isolate, TET^R^, AMP^R^, ATM^R^, CEF^R^	[43]
KP63	Clinical MDR isolate, TET^R,^ CHL^R^, AMP^R,^ ATM^R^	[43]
K24	AcrAB-TolC, Laboratory collection of UNR-MD1, University of Marseille, France	[5]
K2	AcrAB-TolC, Laboratory collection of UNR-MD1, University of Marseille, France	[5]
*Providencia stuartii*		[30]
NEA16	Clinical MDR isolate, AcrAB-TolC	
ATCC29916	Clinical MDR isolate, AcrAB-TolC	
PS2636	Clinical MDR isolate, AcrAB-TolC	
PS299645	Clinical MDR isolate, AcrAB-TolC	
*Pseudemonas aeruginosa*		
PA 01	Reference strains	
PA 124	MDR clinical isolate	[44]

^a^AMP, ATM^R^, CEF^R^, CFT^R^, CHL^R^, FEP^R^, KAN^R^, MOX^R^, STR^R^, TET^R^. Resistance to ampicillin, aztreonam, cephalothin, cefadroxil, chloramphenicol, cefepime, kanamycin, moxalactam, streptomycin, and tetracycline; MDR : Multidrug resistant

### INT colorimetric assay for MIC and MBC determinations

MIC determinations on the tested bacteria were conducted using rapid *p*-iodonitrotetrazolium chloride (INT) colorimetric assay according to described methods [16] with some modifications [19, 20]. The test samples and chloramphenicol were first of all dissolved in DMSO/Mueller Hinton Broth (MHB) or DMSO/7H9 broth. The final concentration of DMSO was lower than 2.5 % and does not affect the microbial growth [21, 22]. The 96- wells microplate were used and the inoculum concentration was 1.5 × 10^6^ CFU/mL [19, 20]. The plates were incubated at 37 °C for 18 h. The assay was repeated thrice. Wells containing adequate broth, bacterial inoculum and DMSO to a final concentration of 2.5 % served as negative control. The MIC of samples was detected after 18 h incubation at 37 °C, following addition (40 μL) of 0.2 mg/mL of INT and incubation at 37 °C for 30 min. Viable bacteria reduced the yellow dye to a pink. MIC was defined as the sample concentration that prevented the color change of the medium and exhibited complete inhibition of microbial growth [16]. The MBC was determined by adding 50 μL aliquots of the preparations, which did not show any growth after incubation during MIC assays, to 150 μL of adequate broth. These preparations were incubated at 37 °C for 48 h. The MBC was regarded as the lowest concentration of extract, which did not produce a color change after addition of INT as mentioned above [19, 20].

## Results and discussion

Compounds tested in this study included five isoflavonoids: atalantoflavone (**2**), bidwillon A (**3**), neocyclomorusin (**4**), 6α-hydroxyphaseollidin (**5**), neobavaisoflavone (**6**) and one flavonoid: sigmoidin I (**1**) (Fig. 1). Their isolation and identification from the bark of *Erythrina sigmoidea* were previously reported [15]. These compounds as well as the crude extracts from the leaves and bark of *Erythrina sigmoidea* were tested for their antibacterial activities on a panel bacterial strains and the results are reported in Tables 2 and 3.

**Table 2:** MICs and MBC (μg/mL) of the crude extracts from *Erythrina sigmoidea* and chlroramphenicol on the panel of tested bacteria

Bacterial strains	Tested plant samples, MIC and MBC (μg/ml) and ratio MBC/MIC								
	*Erythrina sigmoidea* leaves extract			*Erythrina sigmoidea* bark extract			Chloramphenicol		
	MIC	MBC	MBC/MIC	MIC	MBC	MBC/MIC	MIC	MBC	MBC/MIC
*Escherichia coli*									
ATCC8739	64	64	1	16	64	4	4	64	16
AG100	32	256	8	32	128	4	8	>512	na
AG100A	512	1024	2	256	1024	4	4	>512	na
AG100A_TET_	1024	1024	1	256	512	2	32	>512	na
AG102	512	1024	2	128	1024	8	8	>512	na
MC4100	1024	1024	1	512	512	1	32	>512	na
W3110	512	512	1	512	512	1	8	>512	na
*Enterobacter aerogenes*									
ATCC13048	128	256	2	128	1024	8	16	128	8
CM64	1024	>1024	na	1024		na	512	>512	na
EA27	256	256	1	64	128	2	128	>512	na
EA289	1024	>1024	na	512	>1024	na	512	>512	na
EA298	512	512	1	512	1024	2	256	>512	na
EA294	64	512	8	16	128	8	4	32	8
*Enterobacter cloacae*									
ECCI69	1024	>1024	na	1024	>1024	na	256	>512	na
BM47	1024	1024	1	1024	1024	1	512	>512	na
BM67	1024	>1024	na	1024	1024	1	256	>512	na
*Klebsiella pneumoniae*									
ATCC11296	256	256	1	64	512	8	16	128	8
KP55	512	>1024	na	256	>1024	na	64	256	4
KP63	128	>1024	na	16	128	8	128	>512	na
K24	256	512	2	128	>1024	na	16	>512	na
K2	128	1024	8	64	512	8	16	256	
*Providencia stuartii*									
ATCC29916	128	>1024	na	32	128	4	8	128	16
NAE16	128	128	1	32	>1024	na	8	256	32
PS2636	1024	>1024	na	1024	1024	1	64	>512	na
PS299645	512	1024	2	64	128	2	32	>512	na
*Pseudomonas aeruginosa*									
PA01	1024	1024	1	256	256	1	16	256	8
PA124	>1024	>1024	na	>1024	>1024	na	64	|256	4

na: not applicable

**Table 3:** MICs and MBC of compounds from the bark of *Erythrina sigmoidea* on selected bacteria

Bacterial strains	Tested compounds, MIC and MBC (μg/ml) and ratio MBC/MIC																	
	1			2			3			4			5			6		
	MIC	MBC	MBC/MIC	MIC	MBC	MBC/MIC	MIC	MBC	MBC/MIC	MIC	MBC	MBC/MIC	MIC	MBC	MBC/MIC	MIC	MBC	MBC/MIC
*Escherichia coli*																		
ATCC8739	-	-	na	-	-	na	512	-	na	256	-	na	512	-	na	8	-	na
AG100A_TET_	-	-	na	128	-	na	-	-	na	256	-	na	512	-	na	32	-	na
AG102	-	-	na	-	-	na	512	-	na	128	512	4	512	512	1	-	-	na
*Enterobacter aerogenes*																		
ATCC13048	-	-	na	-	-	na	-	-	na	512	-	na	-	-	na	-	-	na
EA289	-	-	na	-	-	na	-	-	na	-	-	na	-	-	na	-	-	na
*Enterobacter cloacae*																		
ECCI69	-	-	na	-	-	na	-	-	na	-	-	na	-	-	na	8	512	64
*Klebsiella pneumoniae*																		
ATCC11296	-	-	na	-	-	na	-	-	na		-		-	-	na	-	-	na
KP55	-	-	na	-	-	na	256	-	na	256	512	2	512	-	na	8	-	na
*Providencia stuartii*																		
ATCC29916	-	-	na	-	-	na	256	-	na	256	-	na	512	-	na	-	-	na
NAE16	-	-	na	-	-	na	-	-	na	256	-	na	512	-	na	8	-	na
*Pseudomonas aeruginosa*																		
PA01	-	-	na	-	-	na	256	-	na	256	-	na	512	-	na	8	-	na
PA124	-	-	na	-	-	na	-	-	na	-	-	na	-	-	na	256	-	na

sigmoidin I (**1**); atalantoflavone (**2**); bidwillon A (**3**); neocyclomorusin (**4**); 6α-hydroxyphaseollidin (**5**); neobavaisoflavone (**6**); (−): MIC or MBC >512 μg/mL; nt: not tested as MIC was >512 μg/mL

Results of the MIC determinations indicate that crude extracts from leaves and bark of this plants were able to inhibit the growth of 26 of the 27 (96.3 %) tested Gram-negative bacteria, and the obtained MIC values ranged from 16 to 1024 μg/mL (Table 2). Compound **1** was not active whilst 2–6 displayed selective activities (Table 3), the MIC values below or equal to 512 μg/mL being noted on 1/12 (8.3 %), 5/12 (41.7 %), 7/12 (58.3 %), 7/12 (58.3 %) and 8/12 (66.7 %) tested bacteria respectively for **2**, **3**, **5**, **6 **and **4**. The lowest MIC value of 16 μg/mL for crude extracts was obtained with the bark extract against *Escherichia coli* ATCC8739, *Enterobacter aerogenes* EA294 and *Klebsiella pneumoniae* KP63. The corresponding value for the tested compounds (8 μg/mL) was obtained with **6 **against *E. coli* ATCC8739, *Enterobacter cloacae* ECCI69, *K. pneumoniae* KP55, *Providencia stuartii* NAE16 and *Pseudomonas aeruginosa* PA01. The antimicrobial activity of a phytochemical (crude extract) has been defined as significant when MIC is below 100 μg/mL, moderate when 100 μg/mL < MIC < 625 μg/mL or low when MIC > 625 μg/mL [4, 23]. On this basis, the crude extracts from *Erythrina sigmoidea* could be considered as promising herbal drug > In fact, MIC values below 100 μg/mL were obtained with leaves and bark extracts respectively against 3/27 (11.1 %) and 10/27 (37.0 %) tested bacteria. Compound **6 ** can also be considered as a good antimicrobial agent, as MIC values below 10 μg/mL were obtained on 5/12 (41.7 %) tested bacteria. Interestingly, the bark extract was more active (lower MIC value) than chloramphenicol on some MDR strains such as *E. aerogenes* EA27, *K. pneumoniae* KP63, highlighting its good antimicrobial potency. Minimal bactericidal concentration (MBC) values below or equal to 1024 μg/mL were also obtained on 18/27 (66.7 %) and 20/27 (74.1 %) tested bacterial strains respectively for leaves and bark extracts. Data from Tables 2 and 3 indicated that some MBC/MIC ratios were below 4, indicating that the studied extracts exerted bactericidal effects on certain Gram negative bacteria [24–26]. However, a keen look of the MICs and MBCs of compounds indicated that they rather exerted bacteriostatic effects (MBC/MIC > 4) [24–26]. It should be noted that the antibacterial spectra of compounds were lower than that of the bark extract. This suggested that a possible synergistic effect between the constituents of this extract could be expected. It should also be noted that the bark extract was not active on the resistant *P. aeruginosa* PA124 strains contrary to the isolated compound **6**. This can either be due to the fact that this active compound (**6**) is less concentrated in the initial crude extract or to the possible interactions with other constituent. Regarding the clinical involvement of MDR bacteria in treatment failures [11, 12, 27, 28], the antibacterial activity of the crude extracts as well as that of compound **6 **could be considered promising. *Pseudomonas aeruginosa* is an important nosocomial pathogen, highly resistant to clinically used antibiotics, leading to substantial morbidity and mortality [29]. MDR Enterobacteriaceae, including *K. pneumoniae, E. aerogenes, E.cloacae* and *P. stuartii* and *E. coli* have also been classified as antimicrobial-resistant organisms of concern in healthcare facilities [11, 12, 30].

To the best of our knowledge, the antibacterial activity of the crude extracts from the *Erythrina sigmoidea* as well as compounds **2**–**6** against MDR bacteria is being reported for the first time. However, the antibacterial activities of compounds belonging to the classes flavonoids and isoflavonoids are well known [31]. In addition, a preliminary antibacterial study of flavonoids from the stem bark of *Erythrina burttii* showed that bidwillon A was active against *E. coli* and *Staphylococcus aureus* [32]. Neobavaisoflavone also displayed antifungal activity against *Aspergillus fumigatus* and *Cryptococcus neoformans* [33]. The present study provides additional information on the antimicrobial potency of neobavaisoflavone (**6**).

## Conclusions

The results of the present study are interesting, taking in account the medical importance of the studied microorganisms. These data provided evidence that the crude extracts from *Erythrina sigmoidea* as well as some of its constituents, and mostly neobavaisoflavone (**6**) could be potential antimicrobial drugs to fight MDR bacterial infections.
